# Sex-specific differences in coronal knee alignment and CPAK distribution in an Austrian population

**DOI:** 10.1007/s00402-025-06125-y

**Published:** 2025-11-24

**Authors:** Amir Koutp, Peter Schieder, Christoph Fetz, Rene Schroedter, Lukas Leitner, Andreas Leithner, Patrick Sadoghi

**Affiliations:** 1 Department of Orthopaedics and Trauma, Medical University of Graz, Graz, Austria; 2 Alps Surgery Institute, Clinique Generale Annecy, Annecy, France; 3 Department of Orthopaedics and Trauma Surgery, Musculoskeletal University Center Munich (MUM), LMU Klinikum, Munich, Germany

**Keywords:** Coronal knee alignment, CPAK, Total knee arthroplasty, Knee phenotype, Osteoarthritis

## Abstract

**Purpose:**

To describe coronal plane alignment patterns and Coronal Plane Alignment of the Knee (CPAK) type distribution in an Austrian population, and to evaluate associations with sex, age, and body mass index (BMI).

**Methods:**

In this retrospective study, 400 knees with complete demographic and radiographic data from standardized long-leg standing radiographs were analyzed. Mechanical lateral distal femoral angle (mLDFA), medial proximal tibial angle (MPTA), arithmetic hip–knee–ankle angle (aHKA), and joint line obliquity (JLO) were measured, and CPAK types were assigned. Statistical analysis included Shapiro–Wilk tests, Welch’s t-test, Chi-square, Fisher’s exact, binary logistic regression, linear regression, and multinomial logistic regression, with significance set at *p* < 0.05.

**Results:**

The cohort comprised 266 females (68%) and 134 males (32%), with a mean age of 68.3 years and mean BMI of 30.4 kg/m². Mean aHKA was − 0.03°, with males showing greater varus alignment than females (− 1.40° vs. +0.67°, *p* = 0.00011). The most frequent CPAK types were I and II (each 21.8%), followed by VI (15.5%), III (15.3%), V (12.5%), and IV (11.3%). CPAK distribution differed by sex (*p* = 0.023) but not by age group. Male sex increased the odds of varus alignment (OR ≈ 2.27, *p* < 0.001) and reduced the odds of valgus alignment (OR ≈ 0.40, *p* < 0.001). BMI was associated with varus alignment in males (*p* = 0.033) but not in females.

**Conclusion:**

In this Austrian cohort, males exhibited greater varus alignment and a distinct CPAK distribution compared to females, while age showed no effect on alignment patterns. BMI predicted varus alignment only in males. These findings provide valuable region-specific CPAK reference data for Austria, supporting individualized alignment strategies in total knee arthroplasty.

## Introduction

Coronal plane alignment of the knee is a key determinant of lower limb biomechanics, influencing tibiofemoral load distribution [1, 2] and the longevity of articular cartilage [3]. Malalignment is implicated in the onset and progression of knee osteoarthritis [1, 4] and is a critical consideration in total knee arthroplasty (TKA) planning [5]. Traditional mechanical hip–knee–ankle (HKA) classifications into varus, neutral, or valgus fail to account for the combined effects of femoral and tibial geometry or for joint line orientation [5].

The Coronal Plane Alignment of the Knee (CPAK) classification addresses these limitations by combining the arithmetic hip–knee–ankle angle (aHKA) with joint line obliquity (JLO) to define nine distinct phenotypes [6]. This approach offers a more nuanced description of native alignment and supports individualized alignment strategies in kinematic and restricted kinematic TKA [6]. Previous studies from Asia, North America, and parts of Europe have shown that CPAK distribution and coronal alignment vary by sex [7–10] and, to a lesser degree, by age, with male knees typically more varus aligned than female knees [8–10]. Higher body mass index (BMI) has been associated with varus alignment, particularly in men [9, 11].

While a large multi-center study has provided initial data [8], further region-specific reference values are needed to inform radiographic assessment and surgical planning within Austria [8, 10]. The aim of this study was to determine the distribution of CPAK types, aHKA, and alignment categories in an Austrian cohort, and to assess their association with sex, age, and BMI. We hypothesized that CPAK distribution and mean aHKA would differ by sex, that age would not significantly influence alignment, and that higher BMI would be associated with greater varus alignment in males.

## Methods

### Patient selection

This retrospective analysis consecutively recruited adults with symptomatic knee osteoarthritis undergoing standardized, weight-bearing, long-leg radiographs at our institution between October 2020 and December 2024. While our group has previously investigated alignment outcomes in TKA comparing kinematic versus mechanical approaches [12], the present study focuses specifically on baseline coronal alignment patterns and CPAK distribution in the Austrian population.

For the current retrospective analysis, knees were included if complete demographic data (sex, age, body mass index [BMI]) and radiographic measurements were available. Only native, untreated knees were analyzed; patients with prior total knee arthroplasty, unicompartmental arthroplasty, corrective osteotomy, periarticular fracture, or radiographs of insufficient quality for accurate measurement were excluded.

### Radiographic measurements

From each image, the mechanical lateral distal femoral angle (mLDFA) and medial proximal tibial angle (MPTA) were measured manually to the nearest degree. The arithmetic hip–knee–ankle angle (aHKA) was calculated as *MPTA − mLDFA*, and the joint line obliquity (JLO) as *MPTA + mLDFA*. The mechanical hip–knee–ankle angle (mHKA) was also computed, and deltaHKA derived as *aHKA − mHKA*.

### CPAK classification and variables

Knees were classified according to the Coronal Plane Alignment of the Knee (CPAK) system, which combines three aHKA categories (varus, neutral, valgus) with three JLO orientations (apex distal, neutral, apex proximal), resulting in nine CPAK types. Sex (male/female), age (continuous and grouped: < 60 years, 60–70 years, >70 years), and body mass index (BMI, kg/m²) were recorded. Alignment was dichotomized as varus (aHKA < 0°) or valgus/neutral (aHKA ≥ 0°). A separate binary variable classified knees as valgus (aHKA > 0°) or not valgus.

### Statistical analysis

All statistical analyses were performed in R version 4.4.1 (*R Foundation*,* Vienna*,* Austria).* Normality of aHKA within each sex was assessed using the Shapiro–Wilk test. Differences in mean aHKA between sexes were evaluated with Welch’s two-sample t-test. Associations between CPAK type and sex or age group were examined using Pearson’s Chi-square and Fisher’s exact tests. Alignment distribution (varus vs. valgus/neutral) across age groups was assessed similarly. Binary logistic regression was used to estimate the effects of sex, age, and BMI on varus versus valgus/neutral alignment and on valgus versus not valgus alignment. Linear regression modeled aHKA as a function of sex, age, BMI, and the BMI × sex interaction, with additional analyses in sex-stratified subsets. Multinomial logistic regression evaluated the influence of sex, age, and BMI on CPAK type assignment. All statistical tests were two-tailed, with p-values < 0.05 considered statistically significant. This was a retrospective consecutive series; therefore, no a-priori sample-size calculation was performed. With the available sample (Female *n* = 266, Male *n* = 134; α = 0.05, two-sided), the study had 96.7% power to detect the observed between-sex difference in aHKA (2.06°, pooled SD 5.10°; Cohen’s *d* 0.40). A difference of at least 1.52° (or 1.76° for 90% power) would have been detectable with 80% (90%) power.

## Results

**Fig. 1:**
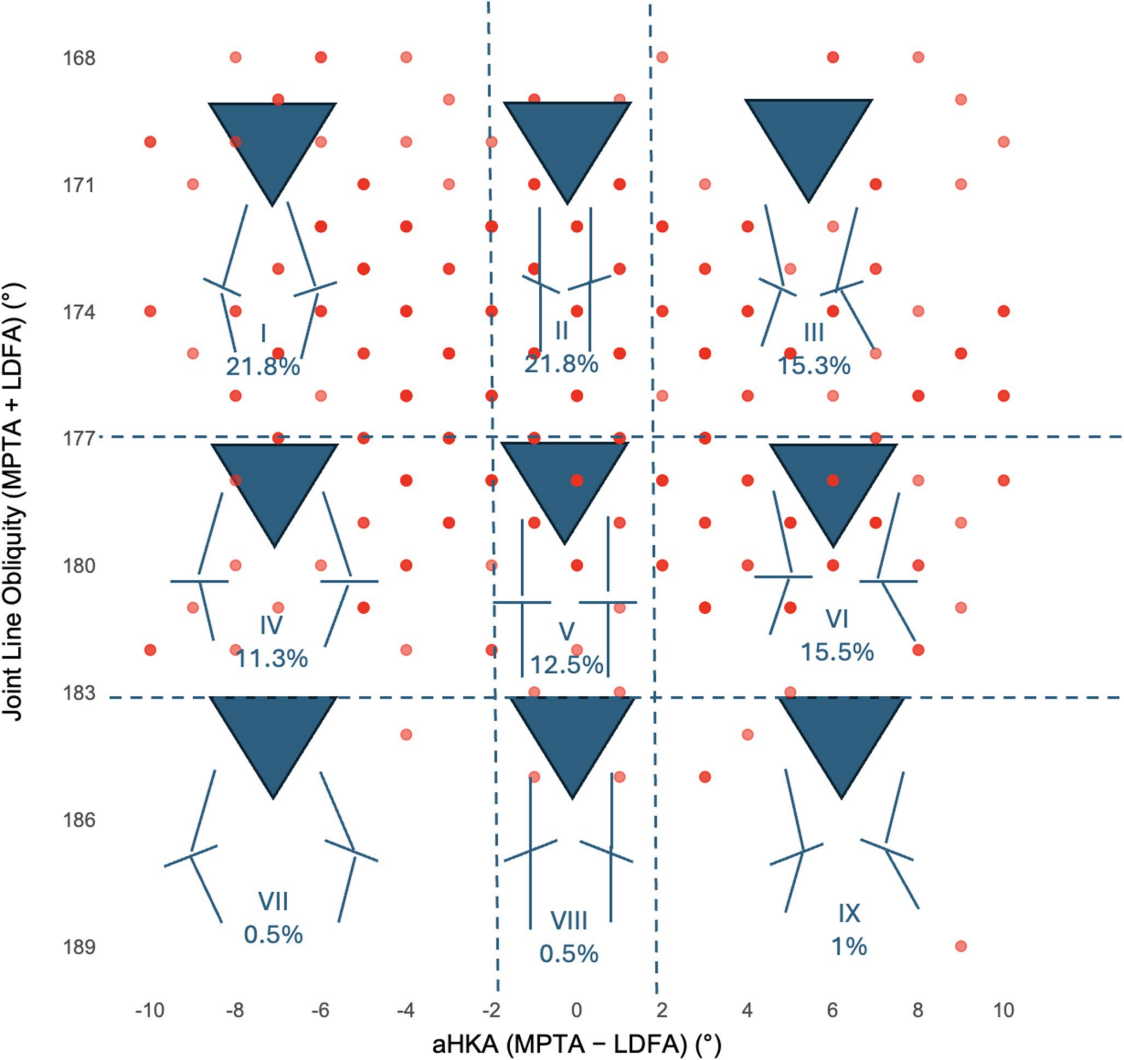
Scatter plot of arithmetic hip–knee–ankle angle (aHKA) versus joint line obliquity (JLO) with Coronal Plane Alignment of the Knee (CPAK) classification boundaries. Dashed vertical lines at − 2° and + 2° aHKA and dashed horizontal lines at 177° and 183° JLO delineate the nine CPAK zones. Roman numerals I–IX indicate the corresponding CPAK type regions. aHKA, arithmetic hip–knee–ankle angle; JLO, joint line obliquity; CPAK, Coronal Plane Alignment of the Knee. Note that some points are overlapping as multiple knees may share the same integer values for aHKA and JLO

A total of 400 knees were analyzed, comprising 266 (68%) from female and 134 (32%) from male subjects. Mean age was 68.3 ± 11.5 years (range 43–89) and mean BMI was 30.4 ± 8.1 kg/m² (range 17.6–50.1) (Table 1). The overall mean aHKA was − 0.03° ± 9.25°, indicating a slight varus tendency. The joint distribution of aHKA and JLO with CPAK boundaries is shown in Fig. 1.

**Table 1:** Descriptive statistics of continuous variables by sex

Variable	Female (*n* = 266)	Male (*n* = 134)	*p*-value (test)
Age (years)	69.21 ± 9.24 (43.00–89.00)	66.59 ± 9.00 (46.00–89.00)	0.00416 (Mann–Whitney U)
BMI (kg/m²)	30.74 ± 5.65 (17.58–50.12)	29.69 ± 5.21 (19.57–46.71)	0.05462 (Mann–Whitney U)
mLDFA (°)	87.53 ± 3.17 (76.00–99.00)	88.33 ± 2.72 (80.00–94.00)	0.00317 (Mann–Whitney U)
MPTA (°)	88.20 ± 3.38 (80.00–99.00)	86.93 ± 3.39 (73.00–95.00)	< 0.001 (Mann–Whitney U)
aHKA (°)	0.67 ± 5.24 (-16.00–16.00)	-1.40 ± 4.82 (-21.00–11.00)	< 0.001 (Welch t-test)
JLO (°)	175.73 ± 3.94 (160.00–189.00)	175.26 ± 3.82 (164.00–185.00)	0.42144 (Mann–Whitney U)

Descriptive statistics of continuous variables by sex

Values are presented as mean ± standard deviation (range). p-values are from Mann–Whitney U tests unless otherwise specified

mLDFA, lateral distal femoral angle; MPTA, medial proximal tibial angle; aHKA, arithmetic hip–knee–ankle angle; JLO, joint line obliquity; SD, standard deviation

**Fig. 2:**
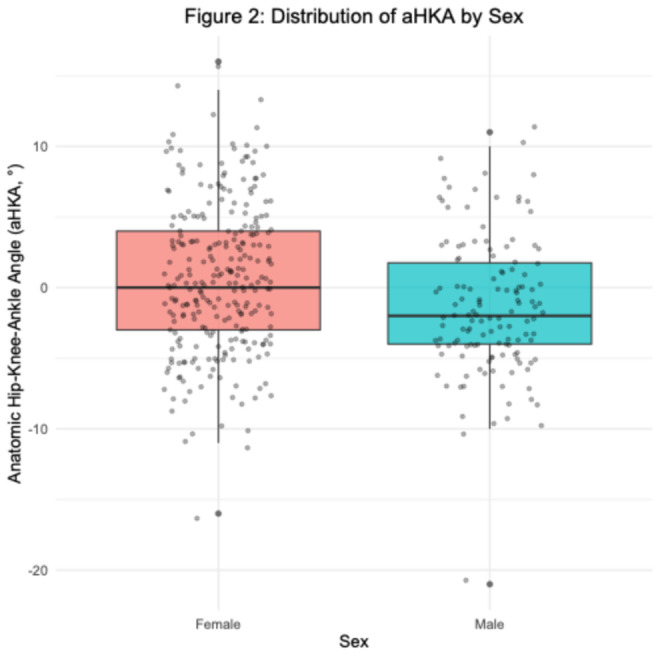
Distribution of anatomic hip–knee–ankle angle (aHKA) by sex

Males demonstrated significantly greater varus alignment than females (mean aHKA − 1.40° vs. +0.67°; mean difference 2.06°; Welch *p* < 0.001; Cohen’s *d* = 0.40; achieved power 96.7%). The distributions by sex are visualized in the box plot (Fig. 2).

Box plots display the median, interquartile range, and outliers, with individual data points overlaid as jittered dots. Males demonstrated significantly greater varus alignment compared with females (mean aHKA − 1.40° vs. +0.67°, *p* < 0.001).

**Fig. 3:**
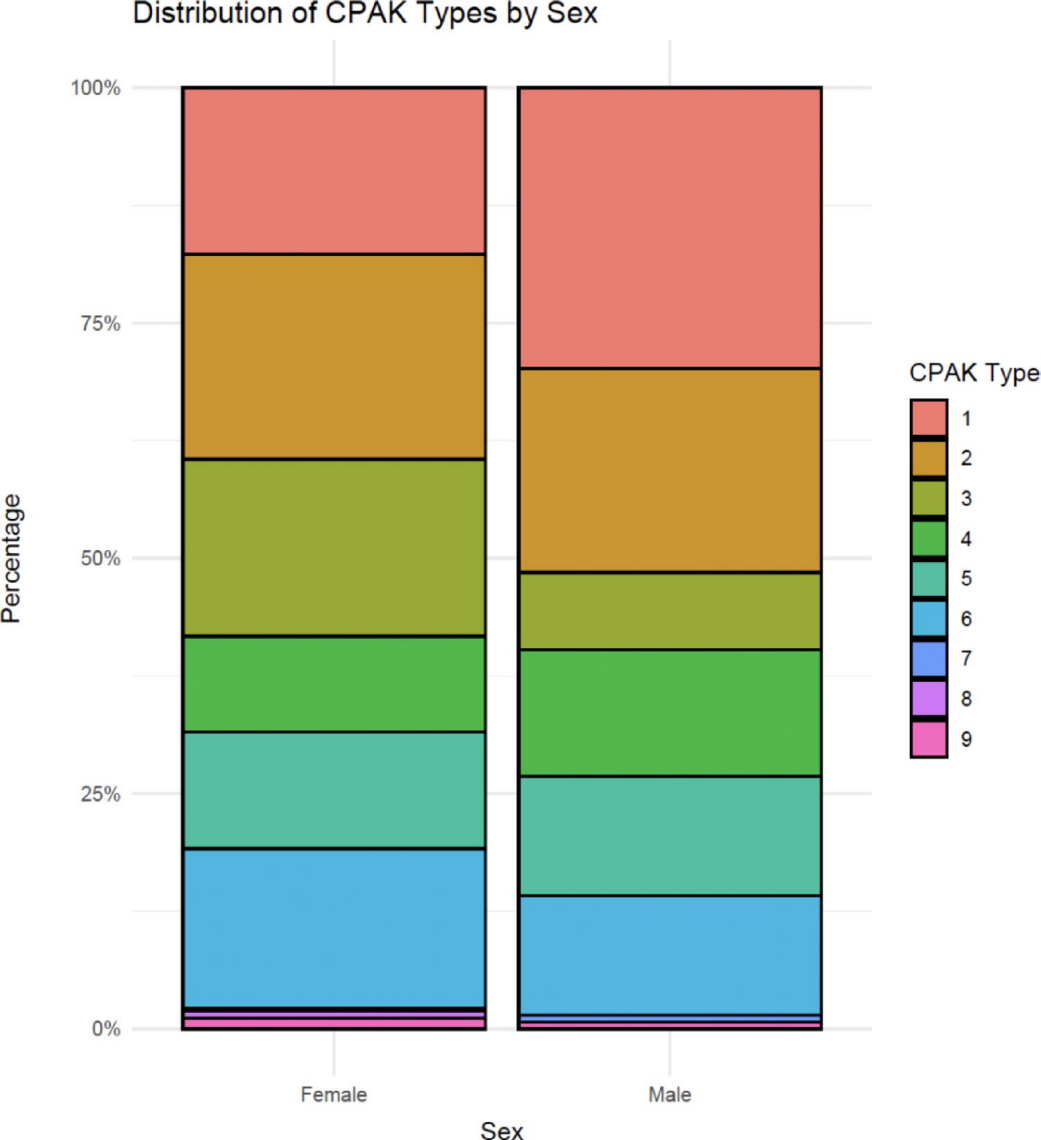
Percentage distribution of Coronal Plane Alignment of the Knee (CPAK) types by sex. Stacked bar charts display the relative proportion of each CPAK type in females (left) and males (right). CPAK, Coronal Plane Alignment of the Knee

The most frequent CPAK types were I and II (each 21.8%), followed by VI (15.5%), III (15.3%), V (12.5%), and IV (11.3%); Types VII–IX each accounted for ≤ 1% of cases. CPAK distribution differed significantly between sexes (*p* = 0.023) but not between age groups (*p* = 0.845) (Table 2). Sex-specific CPAK proportions are displayed in Fig. 3. Alignment category (varus vs. valgus/neutral) was not associated with age group (*p* = 0.173).

**Table 2:** CPAK distribution by sex

CPAK Type	Total (*n*)	Total (%)	Male (*n*)	Male (%)	Female (*n*)	Female (%)	*p*-value (overall sex diff.)
1	87	21.75	40	29.85	47	17.67	0.04382 (Chi-square)
2	87	21.75	29	21.64	58	21.80	
3	61	15.25	11	8.21	50	18.80	
4	45	11.25	18	13.43	27	10.15	
5	50	12.50	17	12.69	33	12.41	
6	62	15.50	17	12.69	45	16.92	
7	2	0.50	1	0.75	1	0.38	
8	2	0.50	0	0.00	2	0.75	
9	4	1.00	1	0.75	3	1.13	

Distribution of Coronal Plane Alignment of the Knee (CPAK) types by sex

Values are n (%). Overall sex difference tested with Pearson’s Chi-square; Fisher’s exact p-value (R simulation) provided for robustness. CPAK, Coronal Plane Alignment of the Knee

In binary logistic regression, male sex was a strong predictor of varus alignment (OR 2.27, 95% CI 1.48–3.48, *p* < 0.001) and inversely associated with valgus alignment (OR 0.40, 95% CI 0.25–0.63, *p* < 0.001). BMI showed a borderline association with varus alignment (*p* = 0.053), while age was not significant in either model (Table 3).

**Table 3:** Logistic regression models with odds ratios (OR) and 95% confidence intervals

Predictor	OR	95% CI (lower)	95% CI (upper)	*p*-value
Intercept	0.079	0.008	0.729	0.02522
C(sex)[T.Male]	2.273	1.467	3.522	< 0.001
BMI	1.038	1.000	1.079	0.05291
Age	1.016	0.993	1.039	0.17538

Binary logistic regression models predicting varus and valgus/neutral alignment

Model 1: Predicting varus alignment (aHKA < 0°). Model 2: Predicting valgus/neutral alignment (aHKA ≥ 0°). Values are odds ratios (OR) with 95% confidence intervals (CI)

aHKA, arithmetic hip–knee–ankle angle; BMI, body mass index

In the full linear regression model, BMI (*p* = 0.060) and age (*p* = 0.066) showed marginal associations with aHKA; sex and BMI × sex interaction were not significant. In sex-stratified models, BMI was significantly associated with greater varus alignment in males (*p* = 0.033) but not in females (Table 3).

Multinomial logistic regression identified male sex as a significant predictor for CPAK Types III (*p* < 0.001), VI (*p* = 0.017), and VIII (*p* < 0.001). BMI showed borderline associations for Types III (*p* = 0.052) and VIII (*p* = 0.050), while age was not a significant determinant of CPAK type.

## Discussion

This study confirms and extends recent findings on CPAK type distribution and coronal alignment parameters in an Austrian population. The principal findings were: (1) male knees were significantly more varus aligned than female knees, with a mean difference in aHKA of approximately 2°; (2) CPAK distribution differed significantly between sexes but not across age groups; and (3) BMI was significantly associated with varus alignment in males, but not in females.

The sex difference in aHKA observed here is consistent with reports from diverse populations. Kubota et al. [13] found that Japanese males demonstrated greater varus alignment than females, a pattern also described in North American [10, 14, 15] and Turkish cohorts [16]. Our finding that Austrian males averaged − 1.40° aHKA compared to + 0.67° in females supports the notion that this morphological difference transcends ethnic [17, 18] and geographic variation [10, 13, 16]. Our results align with the large-scale analysis of 8,739 Austrian knees by Huber et al. [8], who also found that varus morphotypes were more common in males. Our study, using manual measurements in a separate cohort, independently confirms these sex-specific patterns, strengthening the evidence for this demographic trend within the Austrian population. The persistence of this difference after adjusting for age and BMI suggests an underlying anatomical basis, likely reflecting inherent differences in femoral and tibial geometry rather than modifiable factors.

The CPAK distribution in our cohort dominated by Types I and II, followed by Types VI, III, and V shows broad similarity to previous studies [7], albeit with subtle differences. In the Thai arthritic cohort described by Phongpetra et al. [19], Type I predominated (49.3%), similar to our Austrian sample where Types I and II were equally prevalent. When compared to other European populations, our cohort shows notable similarities. For instance, the predominance of CPAK Types I and II mirrors findings in Italian [20] and Spanish [21] cohorts, suggesting a common European distribution pattern. Data from a Turkish population also showed Type I as the most frequent (29.2%) [22]. In contrast, a study of young, healthy Iranian individuals reported CPAK Type V as most common [23], highlighting potential differences between osteoarthritic and non-arthritic populations. The rarity of Types VII–IX in both series reinforces that extreme valgus morphotypes with certain joint line orientations are uncommon across populations [6–8, 20–23]. Huber et al. found that apex proximal joint lines (Types VII–IX) occurred in only 1.3% of cases [8]. Importantly, our finding of significant sex-based variation in CPAK distribution highlights a potential need for sex-specific considerations in surgical planning [7, 8, 10]. In particular, male patients may be overrepresented in CPAK categories associated with varus morphotypes [8, 10], which can influence kinematic or restricted kinematic alignment targets in TKA.

Age was not associated with CPAK type or alignment category in this older adult cohort. This stability from the sixth decade onward contrasts with longitudinal studies reporting progressive varus drift with aging, suggesting that such changes may occur earlier in life or be masked by osteoarthritic deformity in advanced age. Similar stability has been observed in other cross-sectional analyses of older populations [24–26], supporting the view that coronal plane morphology is relatively constant in later adulthood.

BMI emerged as a sex-specific predictor of alignment, with higher BMI linked to greater varus in males but not in females. Ramazanian et al. demonstrated this complex relationship, finding that “at lower BMI, HKA angles were more varus in males than in females. However, at higher BMI, HKA angles were more varus in females than in males [14]. Possible explanations include differences in pelvic width [27], limb loading mechanics [28], and soft tissue laxity [29]. While similar trends have been suggested in other series [8, 9, 30], the inconsistency of prior reports may reflect sample size limitations or differences in BMI distribution between study populations [14, 30].

From a clinical perspective, these findings provide valuable regional reference data for preoperative planning in TKA [6, 8, 31]. For surgeons applying kinematic or restricted kinematic alignment, recognising that Austrian males are more likely to present with varus morphotypes and specific CPAK types can guide implant positioning and ligament balancing [6, 31]. MacDessi et al. demonstrated that ‘across all CPAK types, a greater proportion of KA TKAs achieved optimal balance compared to MA’ with this effect being largest in varus morphotypes (CPAK Types I, II, and IV) [6]. Furthermore, in male patients with elevated BMI, awareness of the potential for greater varus alignment may influence decisions on bone cuts [32], component orientation [31], and postoperative monitoring for uneven implant loading and wear [33].

### Limitations

Nevertheless, certain limitations should be acknowledged. The retrospective design and recruitment from a single Austrian region may limit generalizability to other regions or ethnic groups. Functional outcomes, osteoarthritis grade, and soft tissue laxity were not assessed, although these factors may influence alignment and CPAK distribution. Radiographic measurements were obtained in static, weight-bearing conditions and may not fully capture dynamic alignment during gait. Torsional deformities, such as femoral and tibial torsion, were not assessed and may also contribute to the overall coronal alignment profile.

Finally, the cross-sectional design precludes conclusions regarding causal relationships between BMI, age, and coronal alignment.

## Conclusion

In conclusion, this study of an Austrian population exhibits sex-specific differences in coronal plane alignment and CPAK distribution, with males more likely to present with varus morphotypes and certain CPAK categories. Age does not significantly influence alignment patterns in older adults, while BMI is associated with varus alignment in males only. These findings provide region-specific reference data that may support individualized alignment strategies in TKA and contribute to the broader understanding of coronal alignment variation across populations.

## Data Availability

No datasets were generated or analysed during the current study.
